# Fecal Microbiota Transplantation Improves Biota and Hepatic Metabolism, Promoting Growth in SD Rats Under Hypobaric Hypoxia Exposure

**DOI:** 10.3390/microorganisms14061370

**Published:** 2026-06-20

**Authors:** Shuting Bao, Shengchun Xu, Zhilong Wang, Shatuo Chai, Shuxiang Wang, Dongwen Dai, Xun Wang, Jiaying Lv

**Affiliations:** 1 Academy of Animal and Veterinary Sciences, Qinghai University, Xining 810016, China; ys240905020819@qhu.edu.cn (S.B.); xushengchun2022@163.com (S.X.); 18225922403@139.com (Z.W.); chaishatuo@163.com (S.C.); wangxun513@163.com (X.W.); lvjiaying1123@163.com (J.L.); 2 Key Laboratory of Plateau Grazing Animal Nutrition and Feed Science of Qinghai Province, Qinghai University, Xining 810016, China; 3 Yak Engineering Technology Research Centre of Qinghai Province, Qinghai University, Xining 810016, China; 4 National Yak Technology Innovation Center (Preparatory), Qinghai University, Xining 810016, China

**Keywords:** hypobaric hypoxia, gut microbiota, liver, metabolome, inflammation, growth

## Abstract

Hypobaric hypoxia poses a serious threat to growth and development and can induce pronounced inflammatory responses. These effects are closely associated with the gut microbiota. However, the underlying mechanisms, particularly the role of gut microbiota in regulating hepatic metabolism under chronic hypoxic conditions, remain poorly understood. In this study, SD rats were used as recipients and assigned to three groups: a hypobaric hypoxia group (H), an antibiotic-treated group (HA), and an antibiotic-treated group receiving fecal microbiota transplantation from plateau zokors (HAM). All rats were maintained in a hypobaric hypoxia chamber simulating an altitude of 6000 m for 30 days. Subsequently, growth performance, routine hematological parameters, and multi-omics profiles were evaluated. Compared with the H group, both the HAM and HA groups showed significantly increased average daily gain (ADG) (*p* < 0.05), while the ADG/ADFI ratio was significantly higher in the HAM group than in the H group (*p* < 0.05). Monocyte count (Mon#) and monocyte percentage (Mon%) were significantly higher in the HA group than in both the H and HAM groups (*p* < 0.05). Microbiota analysis revealed significant enrichment of *Lachnospiraceae_NK4A136_group* in the HAM group, whereas *Desulfovibrio* was significantly enriched in the HA group (*p* < 0.05). Fecal metabolomics showed that ursodeoxycholic acid (UDCA) was significantly increased in the HAM group (*p* < 0.05). In the liver metabolome, the anti-inflammatory lipid FAHFA 18:1/20:3 was significantly elevated in the HAM group, whereas pro-inflammatory factors, including uric acid and leukotriene D4, were significantly reduced (*p* < 0.05). Correlation analysis further demonstrated that the abundance of Lachnospiraceae was positively correlated with FAHFA 18:1/20:3 and negatively correlated with uric acid and creatinine (*p* < 0.05). Collectively, these findings indicate that the gut microbiota can modulate gut–liver metabolism, alleviate inflammatory responses, and enhance the adaptation of rats to hypoxic environments. This study provides valuable insights into potential strategies for promoting sustainable animal health and adaptation under hypoxic conditions.

## 1. Introduction

Research has demonstrated that hypoxia poses significant risks to the health and well-being of both humans and animals. For example, hypoxia can disrupt the balance of gut microbiota, leading to metabolic disorders in the intestines and liver and disturbing the enterohepatic circulation. Additionally, it triggers severe inflammatory responses, ultimately resulting in poor health and impaired growth and development [1,2].

The liver is the largest metabolic organ and gland in the human body, acting as the central hub for chemical processing. It regulates numerous physiological and metabolic processes, thereby ensuring both short-term and long-term health [3]. Previous studies have shown that hypoxia induces oxidative stress, which can cause significant damage to the liver. This damage disrupts vital metabolic and immune functions and may even lead to liver fibrosis [4,5]. Thus, the liver plays a critical role in adaptation to hypoxic conditions.

Currently, approximately 140 million people reside at altitudes above 2500 m, and each year, around 40 million individuals ascend to high-altitude regions for various reasons, thereby experiencing hypoxia. Additionally, certain medical conditions, such as sleep apnea–hypopnea syndrome, expose individuals to hypoxic stress. These conditions can lead to liver damage [6]. Furthermore, livestock living in high-altitude regions are also affected by hypoxia, leading to poor health and stunted growth. As a result, the economic development of plateau regions is significantly impacted [7]. Hypoxia is typically associated with dysbiosis of the gut microbiota, which leads to dysfunction of the biota mucus barrier and systemic inflammation [8,9]. The gut microbiota plays a crucial role in the host’s fundamental physiological functions, including digestion and absorption, cellular metabolism, and the oxidative stress response [10,11]. It also affects host ecology and makes it easier to adapt to harsh conditions [12]. Research indicates that the gut microbiota mediates bidirectional interactions within the gut–liver axis [13], and liver diseases are closely related to the state of the gut environment [14,15]. Therefore, improving the health of the gut–liver axis by reshaping the gut microbiota is a potential therapeutic approach for enhancing liver health.

In summary, gut microbiota play a crucial role in regulating the health of the gut and liver through the gut–liver axis under hypoxic conditions. The health of both organs is vital for animals to adapt to low-pressure, low-oxygen environments and improve overall health. However, further investigation is needed to explore the potential of microbiota transplantation for treating hypoxia-induced metabolic abnormalities in the gut and liver. In this study, the donor of the gut microbiota was the *Plateau Zokor*, a small rodent that inhabits underground burrows with low oxygen and high carbon dioxide concentrations [16]. Typically found in alpine meadows at altitudes of 2500–5000 m, the *Plateau Zokor’s* gut is rich in short-chain fatty acid-producing bacteria, such as *Lachnospiraceae*, which help them thrive in high-altitude, low-oxygen environments [17,18]. We hypothesized that transplanting gut microbiota adapted to high-altitude environments can alter metabolic conditions in the gut and liver, reduce the presence of inflammatory substances, and affect blood physiology and growth in rats. This study aims to investigate the effects of gut microbiota on biota and hepatic metabolism under hypoxic conditions and to examine their impact on growth. The findings could provide new insights into the potential use of gut microbiota for treating hypoxic gut–liver diseases and enhancing adaptation to hypoxia.

## 2. Materials and Methods

### 2.1. Experimental Animals and Design

**Figure 1:**
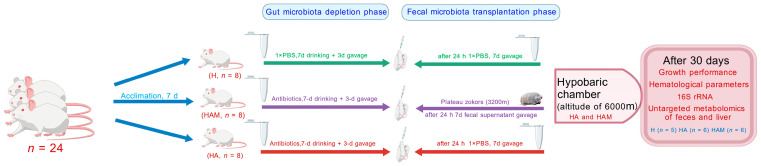
Experimental design and workflow. Created in BioGDP. Shuting Bao. (2026) https://biogdp.com/zh (accessed on 6 June 2026). [19]. H, hypobaric hypoxia group; HA, antibiotic treatment + hypobaric hypoxia group; HAM, antibiotic treatment + plateau zokor fecal microbiota transplantation + hypobaric hypoxia group, Colored arrows are used only to distinguish different experimental procedures and do not indicate additional variables.

Twenty-four 3-week-old male Sprague Dawley (SD) rats weighing 55 ± 10 g were purchased from Vital River Laboratory Animal Technology Co., Ltd. (Beijing, China). The rats were housed in an animal facility in Xining, Qinghai Province, China, at an altitude of approximately 2200 m, under controlled conditions of 22 ± 2 °C, 50–60% relative humidity, and a 12 h light/12 h dark cycle for 7 d of acclimatization. During this period, the rats had free access to a growth diet (Beijing Keao Xieli Feed Co., Ltd., Beijing, China) and water, and the bedding was replaced every 2–3 d. After acclimatization, the rats were randomly assigned to the hypobaric hypoxia group (H group, *n* = 8), the antibiotic pretreatment plus hypobaric hypoxia group (HA group, *n* = 8), and the antibiotic pretreatment plus plateau zokor fecal microbiota transplantation plus hypobaric hypoxia group (HAM group, *n* = 8). The H and HA groups served as controls, whereas the HAM group was designated as the experimental group (Figure 1).

### 2.2. Gut Microbiota Depletion

To deplete the gut microbiota, rats in the HA and HAM groups were provided ad libitum access to a freshly prepared antibiotic cocktail as the sole drinking source for 7 d. The cocktail consisted of vancomycin 0.5 g/L, neomycin 1 g/L, metronidazole 1 g/L, and ampicillin 1 g/L (Sigma-Aldrich, St. Louis, MO, USA) [20]. During the same period, rats in the H group were provided ad libitum access to sterile distilled water. After the 7 d drinking-water treatment, rats in the HA and HAM groups were administered the antibiotic cocktail by oral gavage once daily for 3 consecutive days at a dose of 1 mL/100 g body weight to further deplete the gut microbiota. Rats in the H group received an equal volume of 1× phosphate-buffered saline (PBS; 0.01 M, pH 7.4; Servicebio, Wuhan, China) by oral gavage once daily for 3 consecutive days.

### 2.3. Fecal Microbiota Transplantation

After antibiotic treatment, all rats underwent a 24 h drug withdrawal period, during which they had ad libitum access to sterile distilled water. The donors used for fecal microbiota transplantation were five wild plateau zokors, all of which were captured without injury in Xining, Qinghai Province, China, at an altitude of 3200–3500 m. During the experiment, fresh feces were collected daily and thoroughly homogenized with 1× PBS (Servicebio, Wuhan, China) at a ratio of 1:10 (g/mL). To minimize the potential impact of donor sex on the experimental outcomes, the fecal samples collected from the five donors (including both sexes) were pooled and thoroughly mixed prior to further processing. The resulting fecal suspension was centrifuged at 600× *g* for 15 min at 0 °C, and the supernatant was collected and immediately used for subsequent transplantation. After the withdrawal period, rats in the HAM group were orally gavaged with the plateau zokor fecal microbiota supernatant once daily for 7 consecutive days at a dose of 1 mL/100 g body weight to establish a plateau zokor gut microbiota transplantation model. During the same period, rats in the H and HA groups were orally gavaged with an equal volume of 1× PBS as controls.

### 2.4. Hypobaric Hypoxia Exposure

After fecal microbiota transplantation, all rats were maintained under conventional housing conditions for an additional 2 weeks and then transferred to a hypobaric hypoxia chamber (DYC-300, Guizhou Fenglei Oxygen Chamber Co., Ltd., Guiyang, China) for 30 d of hypoxic exposure. The chamber was used to simulate an altitude of 6000 m, with the temperature maintained at 22 ± 2 °C, relative humidity at 50–60%, and oxygen concentration at 9.2%. During hypoxic exposure, rats had ad libitum access to food and water. Feed intake was continuously monitored for 6 consecutive days after the rats entered the chamber, and body weight was measured repeatedly throughout the experiment to calculate average daily feed intake and average daily gain. On the morning of the last day of hypobaric hypoxia exposure, after fasting, all rats were weighed and anesthetized with urethane (1.0 g/kg, intraperitoneal injection; Sigma-Aldrich, St. Louis, MO, USA) [21]. Approximately 2 mL of blood was collected from the abdominal aorta for subsequent hematological analysis. Gut contents and liver tissues were then collected using sterile instruments, immediately snap-frozen in liquid nitrogen, and stored at −80 °C for subsequent 16S rRNA gene sequencing and untargeted metabolomics analysis. Due to hypobaric hypoxia-induced mortality, the final sample sizes for omics analysis were *n* = 5 for the H group, *n* = 6 for the HA group, and *n* = 6 for the HAM group.

### 2.5. Hematological Analysis

After blood samples were collected, they were immediately analyzed using the BC-2800Vet automated hematology analyzer (Mindray Bio-Medical Electronics Co., Ltd., Shenzhen, China), and detailed records of the red blood cell and white blood cell parameters were obtained.

### 2.6. Gut Microbiota 16S rRNA Gene Sequencing

Microbial total DNA was extracted from gut content samples of SD rats using the CTAB method. DNA integrity was assessed by 1.0% agarose gel electrophoresis, and DNA concentration and purity were determined using a NanoDrop spectrophotometer (Thermo Fisher Scientific, Waltham, MA, USA) or a Qubit fluorometer (Thermo Fisher Scientific, Waltham, MA, USA). The DNA was then diluted to 1 ng/μL with sterile water. The V3–V4 region of the 16S rRNA gene was amplified using barcode-tagged specific primers 341F (5′-CCTAYGGGRBGCASCAG-3′) and 806R (5′-GGACTACNNGGGTATCTAAT-3′). PCR amplification was performed in a 25 μL reaction system containing forward and reverse primers and approximately 5 ng of template DNA. The PCR conditions were as follows: initial denaturation at 94 °C for 5 min; 30 cycles of denaturation at 94 °C for 30 s, annealing at 50 °C for 30 s, and extension at 72 °C for 60 s, followed by a final extension at 72 °C for 7 min. PCR products were checked by 1.0% agarose gel electrophoresis and purified using a MinElute Gel Extraction Kit (Qiagen, Hilden, Germany). The purified products were quantified and pooled in equimolar amounts for paired-end sequencing library construction. PCR amplification, product pooling, purification, library construction, and sequencing were performed by Novogene Bioinformatics Technology Co., Ltd. (Beijing, China) according to standard procedures on the Illumina NovaSeq 6000 platform (Illumina, San Diego, CA, USA).

### 2.7. Untargeted Metabolomics Profiling of Biota Contents and Liver Tissues

Samples were slowly thawed at 4 °C. Gut contents and liver tissue samples from SD rats (50 mg each) were collected and mixed with 400 μL of pre-cooled methanol/acetonitrile solution (1:1, *v*/*v*; methanol and acetonitrile were purchased from Merck, Darmstadt, Germany). The samples were thoroughly vortexed; liver tissue samples were further homogenized and then incubated at −20 °C for 30 min. The samples were subsequently centrifuged at 14,000× *g* for 20 min at 4 °C, and the supernatants were collected and vacuum-dried. Prior to LC–MS analysis, the residues were reconstituted in 100 μL of acetonitrile/water solution (1:1, *v*/*v*; acetonitrile was purchased from Merck, Darmstadt, Germany), vortexed thoroughly, and centrifuged at 14,000× *g* for 15 min at 4 °C. The resulting supernatants were used for injection [22].

Chromatographic separation was performed using an Agilent 1290 Infinity LC ultra-high-performance liquid chromatography (UHPLC) system (Agilent Technologies, Santa Clara, CA, USA) equipped with an ACQUITY UPLC BEH Amide column (1.7 μm, 2.1 mm × 100 mm; Waters, Milford, MA, USA). The column temperature was maintained at 25 °C, the flow rate was 0.5 mL/min, and the injection volume was 2 μL. Mobile phase A consisted of 100% water containing 25 mM ammonium acetate (Sigma-Aldrich, St. Louis, MO, USA) and 25 mM ammonium hydroxide (Sigma-Aldrich, St. Louis, MO, USA), while mobile phase B consisted of 100% acetonitrile (Merck, Darmstadt, Germany). The gradient elution program was as follows: 0–0.5 min, 5% A and 95% B; 7 min, 35% A and 65% B; 8–9 min, 60% A and 40% B; and 9.1–12 min, 5% A and 95% B. During analysis, the samples were maintained at 4 °C in the autosampler.

After UHPLC separation, mass spectrometric detection was performed on an AB TripleTOF 6600 mass spectrometer (AB Sciex, Framingham, MA, USA) equipped with an electrospray ionization (ESI) source in both positive and negative ion modes. The ion source parameters were set as follows: Gas 1, 60; Gas 2, 60; curtain gas, 30 psi; ion source temperature, 600 °C; and ion spray voltage, +5500 V in positive ion mode and −5500 V in negative ion mode. The MS1 scan range was *m*/*z* 60–1000, and the MS/MS scan range was *m*/*z* 25–1000.

### 2.8. Statistical Analysis and Multi-Omics Data Processing

#### 2.8.1. Phenotypic Data Analysis

Average daily gain (ADG), average daily feed intake (ADFI), complete blood count parameters, and other phenotypic data were statistically analyzed using SAS 9.2 software (SAS Institute, Cary, NC, USA). Prior to statistical analysis, the normality of data distribution was assessed using the Shapiro–Wilk test, and homogeneity of variance was evaluated using Levene’s test. For data conforming to a normal distribution and homogeneity of variance, comparisons among the three groups were performed using one-way analysis of variance (ANOVA). When significant differences were detected, Duncan’s multiple-range test was further conducted. Data are presented as the mean ± standard deviation (SD), and *p* < 0.05 was considered statistically significant.

#### 2.8.2. 16S rRNA Gene Sequencing Data Analysis

For 16S rRNA gene sequencing data, gut microbial community composition was analyzed at different taxonomic levels, including the phylum, family, and genus levels. Alpha and beta diversity were calculated and visualized Mothur software (version 1.48.0) and R software (version 4.4.1). Beta diversity was assessed based on unweighted UniFrac distances, and principal coordinate analysis (PCoA) was used to visualize differences in microbial community structure among groups. Permutational multivariate analysis of variance (PERMANOVA) was performed to evaluate intergroup differences in microbial community structure. Linear discriminant analysis effect size (LEfSe) was used to identify significantly enriched bacterial taxa among groups, with the screening thresholds set at LDA score ≥ 4 and *p* < 0.05 [23].

#### 2.8.3. Untargeted Metabolomics Data Analysis

For untargeted metabolomics data, principal component analysis (PCA) and partial least-squares discriminant analysis (PLS-DA) were used to evaluate differences in metabolic profiles among groups. The reliability and predictive ability of the PLS-DA model were evaluated using cross-validation and permutation testing to avoid overfitting. Volcano plots and hierarchical clustering heatmaps were used to visualize differential metabolites. Differential metabolites were identified based on pairwise comparisons using the following criteria: variable importance in projection (VIP) > 1, *p* < 0.05, and fold change *FC* ≥ 1.41 or ≤ 0.71. KEGG pathway enrichment analysis was performed to identify significantly enriched metabolic pathways.

#### 2.8.4. Correlation Analysis Between Gut Microbiota and Metabolites

Spearman correlation analysis was used to evaluate associations between differential gut microbiota and differential metabolites in biota contents and liver tissues.

## 3. Results

### 3.1. The Impact of Microbiota Transplantation on Weight Gain, Feed Intake, and Complete Blood Count

Table 1 presents the effects of fecal microbiota transplantation (FMT) on average daily gain (ADG) and average daily feed intake (ADFI) in rats exposed to hypobaric hypoxia. Both the HAM and HA groups showed significantly higher ADG than the H group (*p* < 0.05), while no significant difference was observed between the HAM and HA groups. No significant differences in ADFI were found among the three groups (*p* > 0.05). The HAM group exhibited a significantly higher ADG/ADFI ratio than the H group (*p* < 0.05), whereas no significant differences were detected between the HA and H groups or between the HAM and HA groups (*p* > 0.05).

As shown in Table 2, there were no significant differences in red blood cell count (RBC), hemoglobin (HGB), hematocrit (HCT), mean corpuscular volume (MCV), mean corpuscular hemoglobin (MCH), mean corpuscular hemoglobin concentration (MCHC), red cell distribution width—coefficient of variation (RDW-CV), red cell distribution width—standard deviation (RDW-SD), platelet count (PLT), mean platelet volume (MPV), platelet distribution width (PDW), or plateletcrit (PCT) among the H, HA, and HAM groups (*p* > 0.05).

As shown in Table 3, no significant differences were observed in white blood cell count (WBC#), neutrophil count (Neu#), lymphocyte count (Lym#), eosinophil count (Eos#), neutrophil percentage (Neu%), lymphocyte percentage (Lym%), or basophil percentage (Bas%) among the three groups (*p* > 0.05). However, compared with the H and HAM groups, the HA group showed a significantly increased monocyte count (Mon#) and monocyte percentage (Mon%) (*p* < 0.05). In addition, eosinophil percentage (Eos%) was significantly lower in the HA group than in the H group (*p* < 0.05), whereas no significant difference was observed between the HA and HAM groups. Basophil count (Bas#) was significantly higher in the HA group than in the HAM group (*p* < 0.05), whereas the H group did not differ significantly from either the HA or HAM group (*p* > 0.05).

### 3.2. The Composition of the Gut Microbiota

**Figure 2:**
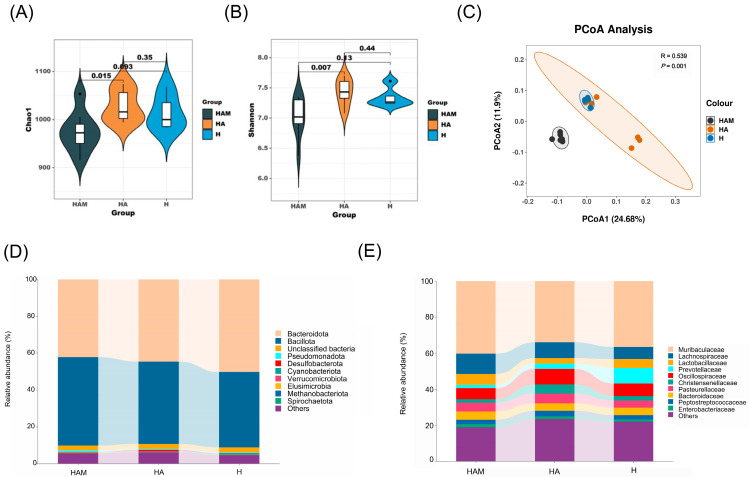
Differential composition of the gut microbiota: (**A**) Chao1 index of different groups. (**B**) Shannon index of different groups. (**C**) PCoA analysis based on the Unweighted-UniFrac distance. (**D**) Differences in bacterial abundance at the phylum level. (**E**) Differences in bacterial abundance at the family level. In subfigures A and B, the white boxes within the violin plots represent the interquartile range (IQR), the horizontal line inside each box indicates the median, and the whiskers indicate the data range. In panel **D**, “Others” includes low-abundance phyla that were not individually displayed, such as Actinomycetota, Campylobacterota, and Planctomycetota. In panel E, “Others” includes low-abundance families that were not individually displayed, such as Oscillospiraceae, Methanobacteriaceae, Clostridiaceae, Tannerellaceae, and Sutterellaceae.

Using amplicon sequence variant (ASV) analysis, a total of 1562 ASVs were identified, including 60 ASVs unique to the HAM group, 76 ASVs unique to the HA group, and 37 ASVs unique to the H group (Figure S1). In terms of alpha diversity indices, the Chao1 index of the HAM group was significantly lower than that of the control groups (*p* < 0.05), with no significant difference between the HA and H groups (*p* > 0.05) (Figure 2A). Regarding the Shannon index, the HAM group was lower than the HA group *(p <* 0.05), with no significant differences observed among the other groups (*p* > 0.05) (Figure 2B). Principal coordinate analysis (PCoA) based on unweighted UniFrac distance revealed significant differences in microbial community composition among the three groups (*p* < 0.05) (Figure 2C). Analysis of the gut content microbiota composition in SD rats showed that, at the phylum level, Bacteroidota and Bacillota were the dominant phyla in the gut content microbiota of both groups (Figure 2D, Table S1). At the family level, Muribaculaceae, Lachnospiraceae, and Lactobacillaceae were the predominant bacterial families (Figure 2E). At the genus level, the relative abundances of *Prevotella*, *Prevotella_9*, and *Limosilactobacillus* in group H were significantly higher than those in the HAM and HA groups (*p* < 0.05). *Lachnospiraceae_NK4A136_group* and *[Eubacterium]_ruminantium_group* were significantly enriched in the HAM group (*p* < 0.05). Meanwhile, the abundances of *Christensenellaceae_R-7_group*, *NK4A214_group*, and *Desulfovibrio* in group HA were significantly higher than those in the HAM group (*p* < 0.05; Table S2).

**Figure 3:**
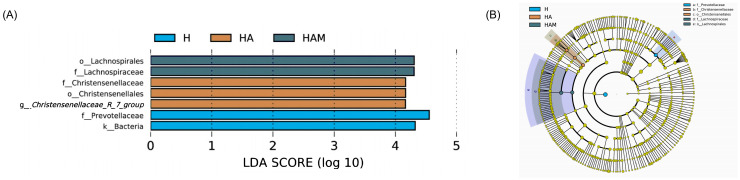
Linear discriminant analysis effect size was performed to determine the difference in abundance; the threshold of the LDA score was 4.0: (**A**) The histogram of the LDA value. (**B**) Phylogenetic tree diagram.In panel B, each circle represents a taxonomic unit from phylum to genus levels. Yellow dots indicate taxa with no significant differences among groups, whereas colored dots indicate taxa significantly enriched in the corresponding group.

We utilized Linear Discriminant Analysis Effect Size to further identify biomarkers. o_ Lachnospirales and f_ Lachnospiraceae were abundant in the HAM group; o_ Christensenellales, f_ Christensenellaceae, and g_ *Christensenellaceae_R-7_group* were abundant in the HA group; and f_ Prevotellaceae and k_ bacteria were abundant in the H group. (Figure 3A,B and Figure S2).

### 3.3. Fecal and Liver Metabolism

**Figure 4:**
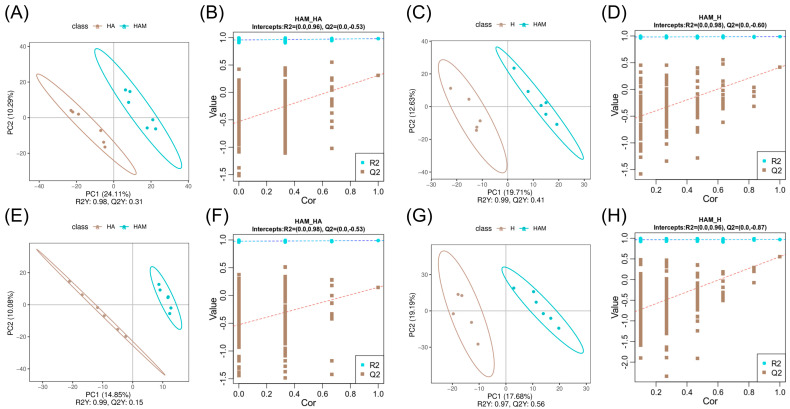
PLS-DA plot of fecal and liver metabolites: (**A**,**B**) Fecal metabolites comparing the HAM and HA groups. (**C**,**D**) Fecal metabolites comparing the HAM vs. H groups. (**E**,**F**) Liver metabolites comparing the HAM vs. HA groups. (**G**,**H**) Liver metabolites comparing the HAM vs. H groups. In panels B, D, F, and H, the blue dashed line represents the R^2^ regression line, while the red dashed line represents the Q^2^ regression line obtained from permutation testing.

The partial least squares discriminant analysis (PLS-DA) illustrated the differences in fecal and liver metabolite composition between the HAM and HA groups (Figure 4A,B,E,F), as well as between the HAM and H groups (Figure 4C,D,G,H). The R^2^ value exceeded the Q^2^ value, and the Q^2^ regression line intercepts the y-axis below zero, indicating that the model was not overfitted.

**Figure 5:**
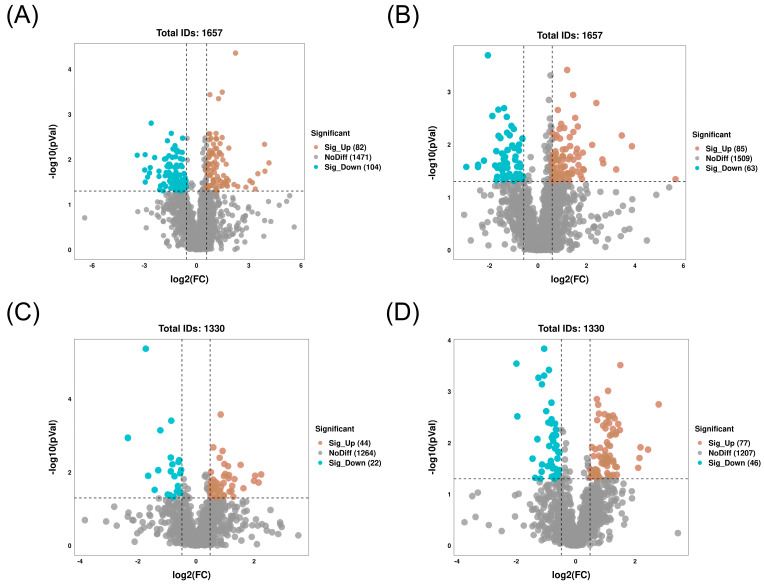
Differences in fecal and liver metabolites: (**A**) Volcano plot of fecal metabolites comparing the HAM and HA groups. (**B**) Volcano plot of fecal metabolites comparing the HAM vs. H groups. (**C**) Volcano plot of liver metabolites comparing the HAM vs. HA groups. (**D**) Volcano plot of liver metabolites comparing the HAM vs. H groups.The vertical dashed lines indicate the fold-change threshold (|log2*FC*| = 0.5), and the horizontal dashed line indicates the significance threshold (*p* = 0.05).

The volcano plot results indicate that, in terms of fecal metabolite analysis, the HAM group exhibited 186 differential metabolites compared to the HA group and 148 differential metabolites compared to the H group (Figure 5A,B). In terms of liver metabolite analysis, the HAM group exhibited 66 differential metabolites compared to the HA group and 123 differential metabolites compared to the H group (Figure 5C,D).

**Figure 6:**
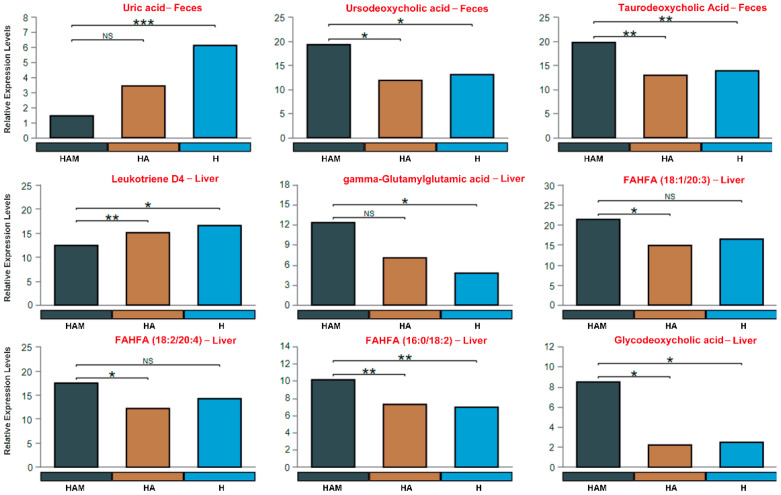
Expression levels of key differential metabolites in the liver and feces. *, *p* < 0.05; **, *p* < 0.01; ***, *p* < 0.001; NS, *p* > 0.05.

Clustering heatmap analysis of differential fecal metabolites revealed that the relative abundances of tauroursodeoxycholic acid dihydrate, ursodeoxycholic acid, taurodeoxycholic acid, emetine, and LPC 18:2 were elevated in the HAM group compared with the HA and H groups. Among them, ursodeoxycholic acid, taurodeoxycholic acid, and emetine were significantly enriched in the HAM group (*p* < 0.05). Conversely, the relative abundances of uric acid, purine, indole-3-acrylic acid, and cyclic ADP-ribose were reduced in the HAM group. In particular, uric acid levels were significantly lower than those in the H group (*p* < 0.05), although no significant difference was detected between the HAM and HA groups (*p* > 0.05) (Figure 6 and Figure S3).

Clustering heatmap analysis of hepatic metabolites revealed that the levels of PC (16:0/20:5), PC (20:5/20:5), PC (18:3/20:5), gamma-glutamylglutamic acid, taurodeoxycholic acid, glycodeoxycholic acid, FAHFA (18:2/20:4), FAHFA (18:1/20:3), and orotic acid were elevated in the HAM group compared with the control group. Among these metabolites, gamma-glutamylglutamic acid was significantly increased in the HAM group relative to the H group (*p* < 0.05), whereas no significant difference was observed between the HAM and HA groups (*p* > 0.05). Likewise, the levels of FAHFA (18:2/20:4) and FAHFA (18:1/20:3) were significantly higher in the HAM group than in the HA group (*p* < 0.05), but were comparable to those in the H group (*p* > 0.05). Furthermore, PC (16:0/20:5) and glycodeoxycholic acid were significantly enriched in the HAM group compared with both the H and HA groups (*p* < 0.05). In contrast, the levels of creatinine, D-proline, DL-o-tyrosine, PC (20:1/20:1), L-carnitine, (5-L-glutamyl)-L-amino acid, and leukotriene D4 were reduced in the HAM group. Notably, leukotriene D4 was significantly decreased in the HAM group relative to both the HA and H groups (*p* < 0.05, Figure 6 and Figure S4).

KEGG pathway enrichment analysis demonstrated that the differential fecal metabolites between the HAM and HA groups were primarily associated with caffeine metabolism, biosynthesis of plant secondary metabolites, D-glutamine and D-glutamate metabolism, alanine, aspartate, and glutamate metabolism, and Microbial metabolism in diverse environments (*p* < 0.05, Figure S5a). In comparison with the H group, the differential metabolites in the HAM group were mainly involved in the calcium signaling pathway, pentose phosphate pathway, and beta-alanine metabolism (*p* < 0.05, Figure S5b).

In the liver, differential metabolites between the HAM and HA groups were significantly associated with pyruvate metabolism and valine, leucine, and isoleucine biosynthesis (*p* < 0.05, Figure S6a). Moreover, compared with the H group, the differential metabolites in the HAM group were mainly involved in arginine and proline metabolism, aminobenzoate degradation, and porphyrin and chlorophyll metabolism (*p* < 0.05, Figure S6b).

**Figure 7:**
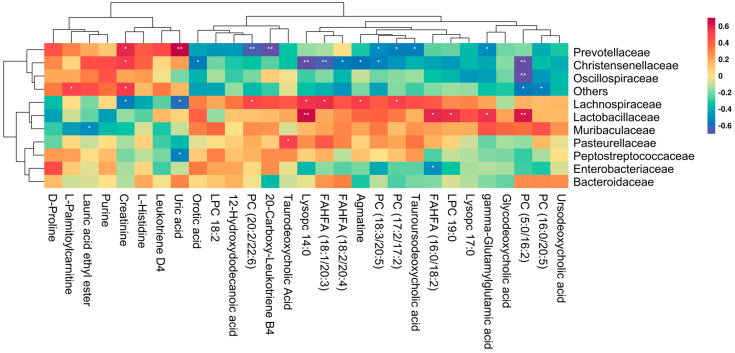
The Spearman correlation analysis between family and differential metabolites in the gut and liver. *, *p* < 0.05; **, *p* < 0.01.

We utilized Spearman correlation coefficients to analyze the relationship between gut and liver metabolites and the gut microbiota (Figure 7). This analysis aimed to elucidate the connections between differentially enriched bacteria and differential metabolites, exploring the impact of microbiota on metabolism. The results indicated that Lachnospiraceae showed a significant positive correlation with PC (17:2/17:2), agmatine, and FAHFA (18:1/20:3) and a significant negative correlation with uric acid and creatinine (*p* < 0.05). Additionally, Lactobacillaceae exhibited a significant positive correlation with PC (5:0/16:2), gamma-glutamylglutamic acid, and FAHFA (16:0/18:2)(*p* < 0.05).

## 4. Discussion

Appropriate body weight gain is an important indicator for evaluating animal health status and environmental adaptability. In high-altitude hypoxic environments, the gut microbiota is closely associated with host growth and development, energy metabolism, and adaptation to hypoxic stress [2,24]. Previous studies have shown that fecal microbiota transplantation (FMT) can improve growth performance in pigs at different growth stages and modulate the composition and metabolic functions of the hindgut microbiota [25]. In the present study, under simulated hypobaric hypoxic conditions, the average daily gain (ADG) of both the HAM and HA groups was significantly higher than that of the H group, whereas no significant difference in average daily feed intake (ADFI) was observed among the three groups. These findings suggest that antibiotic pretreatment and FMT intervention may promote body weight gain in rats without markedly affecting feed intake. Further analysis showed that the ADG/ADFI ratio was significantly higher in the HAM group than in the H group, indicating a potential improvement in feed utilization efficiency. However, no significant differences in ADG or ADG/ADFI were observed between the HAM and HA groups, suggesting that these changes cannot be fully attributed to the transplanted microbiota itself. Antibiotic-induced perturbation of the gut microbiota and its subsequent remodeling may also influence host metabolism. Previous studies have demonstrated that antibiotic-mediated disruption of gut microbial homeostasis can alter host metabolism in mice and is associated with an increased risk of obesity [26,27]. Therefore, the higher ADG observed in the HA group, despite a similar feed intake to that of the H group, may be related to changes in nutrient utilization efficiency, metabolic efficiency, or patterns of energy expenditure caused by antibiotic pretreatment-induced gut microbiota depletion.

Complete blood count (CBC) mainly involves the analysis of three blood components: red blood cells (RBCs), white blood cells (WBCs), and platelets (PLTs). Alterations in any of these components may lead to changes in various physiological and metabolic processes and functions [28]. When animals are exposed to high-altitude environments, their hematological and biochemical parameters vary with altitude. It has been demonstrated that dwarf recessive white chickens raised at high altitude exhibit higher RBC counts and hematocrit values than their counterparts raised at low altitude [29]. In the present study, CBC parameters were measured in SD rats exposed to hypobaric hypoxic conditions. The results showed that RBC counts in the HAM, HA, and H groups were higher than the reference range for routine hematological parameters in low-altitude SD rats (6.06–9.31 × 10^12^/L) [30]. A marked increase in RBC count can increase blood viscosity, thereby imposing a greater burden on the heart and contributing to circulatory disorders involving the pulmonary and cerebral circulation [31]. Compared with the HA and H groups, the RBC count in the HAM group decreased by 4.954% and 2.996%, respectively. This suggests that, after microbiota transplantation, blood viscosity in the HAM group was lower than that in the H and HA groups, and oxygen transport capacity was improved. Neutrophils (Neu) are a subtype of white blood cells (WBCs) and represent the most abundant leukocyte population [32]. Monocytes (Mon), the largest blood cells, primarily function in phagocytosis and play key roles in resisting infection, regulating the immune system, repairing tissue injury, defending against pathogen invasion, and conferring disease immunity. They can also accumulate extensively at inflammatory sites, leading to increased numbers in peripheral circulation [33]. Compared with the H and HAM groups, the Neu# in the HA group increased by 7.855% and 51.27%, respectively. In addition, both the Mon# and Mon% in the HA group were significantly higher than those in the H and HAM groups. These findings indicate that antibiotic-mediated depletion of the gut microbiota in the HA group, combined with simulated hypobaric hypoxic exposure, may have induced an immune stress response.

The role of the gut microbiota in nutrient absorption, metabolic regulation, maintenance of host immunity, and intestinal barrier homeostasis has been widely recognized [34]. In the present study, although Bacillota and Bacteroidota were the dominant phyla in the HAM, HA, and H groups, the Bacillota/Bacteroidota ratio was highest in the HAM group. Previous studies have shown that a decreased Bacillota/Bacteroidota ratio is associated with diseases such as inflammatory bowel disease, depression, and Alzheimer’s disease, which may be related to reduced short-chain fatty acid production and immune-inflammatory responses induced by protein-derived metabolites [35,36].

At the family level, Lachnospiraceae was enriched in the HAM group. Members of this family are important short-chain fatty acid-producing bacteria and are also involved in bile acid transformation [37,38]. Through these functions, they contribute to host metabolism, immune regulation, intestinal barrier maintenance, and resistance to intestinal pathogen colonization [39]. Previous studies have shown that reduced Lachnospiraceae abundance is associated with impaired health status, whereas its enrichment is related to the alleviation of high-altitude-induced myocardial hypertrophy and pulmonary hypertension [40]. Therefore, the increased abundance of Lachnospiraceae in the HAM group may represent one of the key microbial features underlying the beneficial effects of FMT.

To further investigate the role of the gut microbiota, the present study analyzed the top 20 most abundant genera. The results showed that *Lachnospiraceae_NK4A136_group* was enriched in the HAM group, which was consistent with the enrichment of Lachnospiraceae at the family level. This further suggests that fecal microbiota transplantation derived from plateau zokors may promote the colonization or expansion of related beneficial bacterial taxa. In addition, enrichment of *Desulfovibrio* was observed in the HA group. *Desulfovibrio* is a sulfate-reducing bacterium that can produce lipopolysaccharide and has been associated with inflammation and metabolic disorders [41,42]. Previous studies have reported that, under hypoxic conditions, *Desulfovibrio* can induce γδ T-cell proliferation, thereby aggravating intestinal injury [43,44,45]. The enrichment of *Desulfovibrio* in the HA group may indicate increased susceptibility to intestinal or immune dysregulation following antibiotic treatment.

High-altitude hypoxia can enhance oxidative stress and inflammatory responses and induce alterations in inflammation-related metabolites [46]. Bile acids are primarily synthesized from cholesterol in the liver and transported through the enterohepatic circulation. In addition to participating in lipid digestion, they also act as signaling molecules involved in the regulation of glucose, lipid, and energy metabolism [47,48]. A bidirectional regulatory relationship exists between the gut microbiota and bile acids; therefore, hypoxia-induced dysregulation of bile acid metabolism may further affect gut microbiota composition and participate in the regulation of intestinal or hepatic inflammatory responses [49]. In the present study, fecal ursodeoxycholic acid (UDCA) levels were significantly increased in the HAM group. UDCA is a naturally occurring bile acid with anti-inflammatory, antioxidant, and immunomodulatory properties, and its protective effects in liver and intestinal diseases have received considerable attention [50,51,52]. The increased UDCA level in the HAM group may indicate a certain improvement in gut–liver axis-related bile acid metabolism and may contribute to alleviating hypoxia-induced oxidative stress and inflammatory responses.

In addition to alterations in bile acid metabolism, several inflammation-related metabolites in the liver were also changed in the HAM group. Among them, the levels of lipid metabolites with anti-inflammatory properties, such as fatty acid esters of hydroxy fatty acids (FAHFAs), increased, whereas the levels of metabolites associated with inflammatory responses, including uric acid and leukotriene D4, decreased. FAHFAs are a class of bioactive lipids with metabolic regulatory and anti-inflammatory activities [53]. Correlation analysis showed that Lachnospiraceae was significantly positively correlated with hepatic FAHFA 18:1/20:3, suggesting that the increased abundance of Lachnospiraceae in the HAM group may be associated with enhanced hepatic anti-inflammatory lipid metabolism. As the end product of purine metabolism, uric acid often accumulates under hypoxic or systemic inflammatory conditions and may contribute to the development and progression of pulmonary hypertension through pro-inflammatory and vascular effects [54,55,56,57,58]. In the present study, Lachnospiraceae was significantly negatively correlated with hepatic uric acid and creatinine. From the perspective of microbiota–liver metabolite associations, this finding further suggests that inflammation- or metabolic stress-related products may be reduced in the HAM group. Taken together, the alterations in bile acids and hepatic inflammation-related metabolites in the HAM group, together with the correlations between Lachnospiraceae and metabolites such as FAHFAs and uric acid, indicate that fecal microbiota transplantation derived from plateau zokors may partially alleviate hypoxia-induced inflammatory–metabolic dysregulation by modulating the gut microbiota and gut–liver axis-related metabolic processes.

## 5. Conclusions

In conclusion, through FMT, the gut microbiota modulated biota and hepatic metabolism and bile acid metabolism and improved the growth and development of rats. It produced more anti-inflammatory substances and reduced the expression of pro-inflammatory substances, thereby potentially alleviating hypoxia-induced biota and hepatic inflammatory responses. However, these findings are preliminary and require further exploration to confirm and apply them in practice. Nevertheless, this study reveals the crucial role of gut microbiota in regulating biota and hepatic metabolism and improving growth and development under hypoxic conditions, providing new insights for hypoxia adaptation research.

## Figures and Tables

**Table 1:** The effects of FMT on ADG and ADFI in rats exposed to hypobaric hypoxia.

Group	ADG (g)	ADFI (g)	ADG/ADFI
HAM	2.30 ± 0.11 ^a^	20.00 ± 1.45	0.11 ± 0.00 ^a^
HA	2.22 ± 0.32 ^a^	21.97 ± 1.47	0.10 ± 0.02 ^ab^
H	1.56 ± 0.04 ^b^	20.64 ± 1.37	0.08 ± 0.00 ^b^
F-value	5.109	2.928	5.087
*p*-value	0.019	0.052	0.020

Note: ADG, average daily gain; ADFI, average daily feed intake. The ADG/ADFI ratio is the body weight gain per unit of feed consumed. Data in the same column with different superscript letters indicate a statistically significant difference (*p* < 0.05).

**Table 2:** The effects of FMT on red blood cells in rats exposed to hypobaric hypoxia.

Item	HAM	HA	H	F-Value	*p*-Value
RBC/(10^12^/L)	10.36 ± 0.85	10.68 ± 0.79	10.90 ± 0.77	0.765	0.479
HGB/(g/L)	269.11 ± 9.49	275.38 ± 6.52	266.00 ± 11.73	1.865	0.182
HCT/(%)	67.27 ± 2.80	68.56 ± 2.82	66.34 ± 2.92	1.013	0.382
MCV/(fL)	65.16 ± 3.38	64.53 ± 5.61	61.04 ± 3.47	1.532	0.242
MCH/(pg)	26.09 ± 1.53	25.90 ± 1.84	24.50 ± 1.54	1.614	0.225
MCHC/(g/L)	400.22 ± 7.45	402.00 ± 11.06	401.20 ± 3.96	0.095	0.911
RDW-CV/(%)	22.36 ± 1.82	21.80 ± 1.18	22.68 ± 1.14	0.604	0.557
RDW-SD/(fL)	64.27 ± 6.28	62.59 ± 5.60	62.28 ± 5.02	0.261	0.773
PLT/(10^9^/L)	337.89 ± 114.72	319.25 ± 71.26	378.60 ± 68.88	0.650	0.533
MPV/(fL)	6.46 ± 0.39	6.60 ± 0.54	6.46 ± 0.39	0.699	0.509
PDW/(%)	6.77 ± 0.46	16.49 ± 0.43	16.52 ± 0.38	0.177	0.839
PCT/(%)	0.23 ± 0.079	0.21 ± 0.051	0.24 ± 0.044	0.446	0.647

Note: RBC, red blood cell count; HGB, hemoglobin; HCT, hematocrit; MCV, mean corpuscular volume; MCH, mean corpuscular hemoglobin; MCHC, mean corpuscular hemoglobin concentration; RDW-CV, red blood cell distribution width—coefficient of variation; RDW-SD, red blood cell distribution width—standard deviation; PLT, platelet count; MPV, mean platelet volume; PDW, platelet distribution width; PCT, plateletcrit.

**Table 3:** The effects of FMT on white blood cells in rats exposed to hypobaric hypoxia.

Item	HAM	HA	H	F-Value	*p*-Value
WBC#/(10^9^/L)	7.81 ± 3.33	8.18 ± 2.08	8.15 ± 1.56	0.050	0.952
Neu#/(10^9^/L)	2.36 ± 1.19	3.57 ± 1.84	3.31 ± 0.71	1.730	0.204
Lym#/(10^9^/L)	5.01 ± 2.63	3.58 ± 1.33	4.39 ± 1.28	1.116	0.348
Mon#/(10^9^/L)	0.37 ± 0.30 ^b^	0.91 ± 0.51 ^a^	0.36 ± 0.27 ^b^	5.131	0.017
Eos#/(10^9^/L)	0.027 ± 0.02	0.015 ± 0.01	0.034 ± 0.02	2.547	0.105
Bas#/(10^9^/L)	0.032 ± 0.03 ^b^	0.098 ± 0.07 ^a^	0.060 ± 0.02 ^ab^	4.041	0.034
Neu%	33.01 ± 13.16	42.00 ± 14.15	40.70 ± 6.70	1.245	0.311
Lym%	61.86 ± 14.33	44.95 ± 17.71	53.28 ± 5.57	2.906	0.079
Mon%	4.39 ± 3.17 ^b^	11.65 ± 7.68 ^a^	4.84 ± 4.25 ^b^	4.325	0.028
Eos%	0.38 ± 0.11 ^ab^	0.21 ± 0.15 ^b^	0.44 ± 0.21 ^a^	4.368	0.027
Bas%	0.37 ± 0.27	1.19 ± 1.00	0.74 ± 0.25	3.449	0.053

Note: WBC, white blood cell count; Neu, neutrophil; Lym, lymphocyte; Mon, monocyte; Eos, eosinophil; Bas, basophil; #, absolute count; %, percentage. Data in the same column with different superscript letters indicate a statistically significant difference (*p* < 0.05).

## Data Availability

The original data of 16s rRNA gene sequencing have been deposited in NCBI under accession number PRJNA767340. The metabolomics data have been added to the China National GenBank Database (CNGBdb) under accession number CNP0003641.

## Supplementary Materials

The following supporting information can be downloaded at: https://www.mdpi.com/article/10.3390/microorganisms14061370/s1, Figure S1: Venn diagram based on ASVs clustering; Figure S2: Clustering heatmap of genus level; Figure S3: Clustering heatmap of differential metabolites in fecal metabolites; Figure S4: Clustering heatmap of differential metabolites in liver metabolites; Figure S5: (a) KEGG enrichment bubble plot of fecal metabolites in HAM vs. HA. (b) KEGG enrichment bubble plot of liver metabolites HAM vs. H; Figure S6: (a) KEGG enrichment bubble plot of liver metabolites in HAM vs. HA. (b) KEGG enrichment bubble plot of liver metabolites in HAM vs. H. Table S1. Relative abundance of gut microbiota at the phylum level. Table S2. Relative abundance of gut microbiota at the genus level.

## Abbreviations

The following abbreviations are used in this manuscript:

ASV	Amplicon Sequence Variant
ANOVA	Analysis of Variance
PCA	Principal Component Analysis
PCoA	Principal Coordinate Analysis
LEfSe	Linear Discriminant Analysis Effect Size
LDA	Linear Discriminant Analysis
PERMANOVA	Permutational Multivariate Analysis of Variance
PLS-DA	Partial Least Squares Discriminant Analysis
VIP	Variable Importance in Projection
FC	Fold Change
