# Supplementary material for: Fecal Microbiota Transplantation Improves Biota and Hepatic Metabolism, Promoting Growth in SD Rats Under Hypobaric Hypoxia Exposure
Source: Microorganisms. 2026 Jun 20;14(6):1370. doi: 10.3390/microorganisms14061370 (PMC13305931; doi:10.3390/microorganisms14061370)
Supplement: Supplementary file 1 [file microorganisms-14-01370-s001.zip › microorganisms-4307445-supplementary.pdf]

Additional file. Figure S1. Venn diagram based on ASVs clustering.

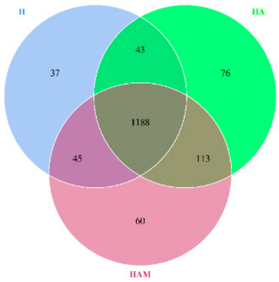

Additional file. Figure S2. Clustering heat map of genus level.

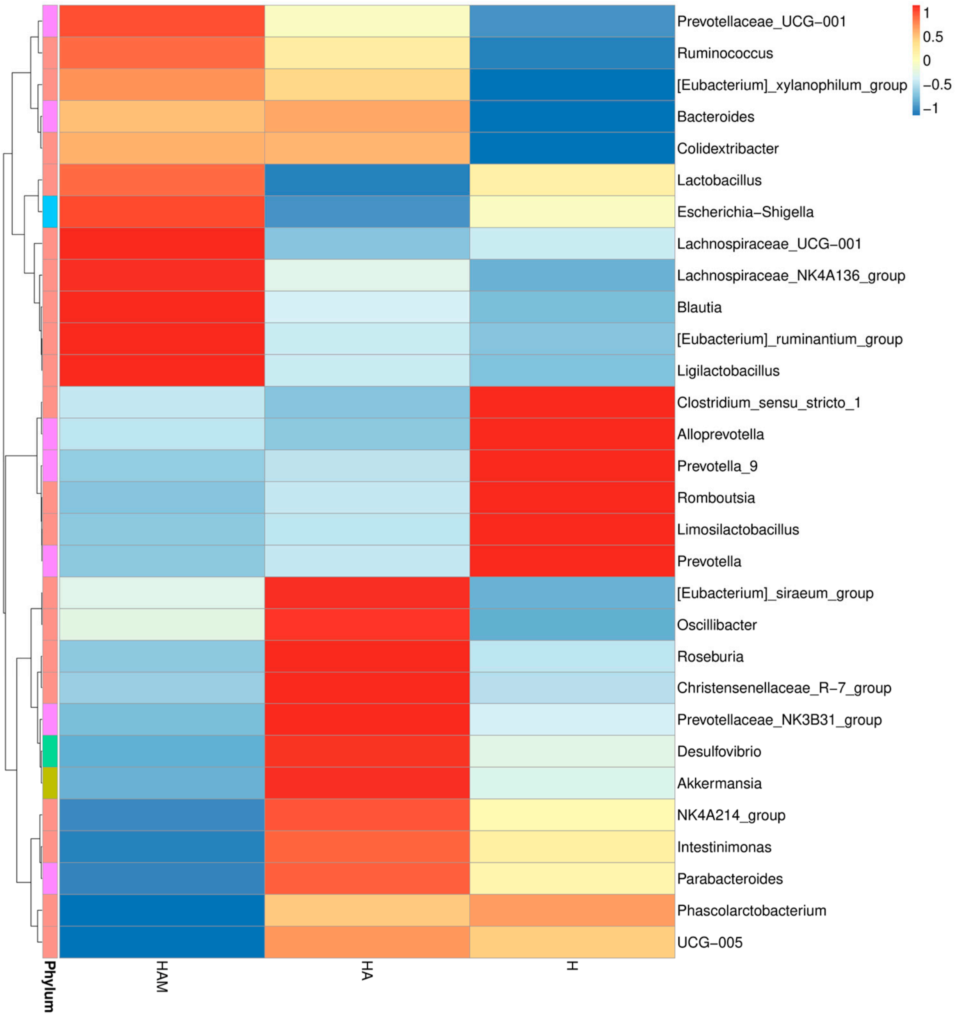

Additional file. Figure S3. Clustering heat map of differential metabolites in fecal metabolites.

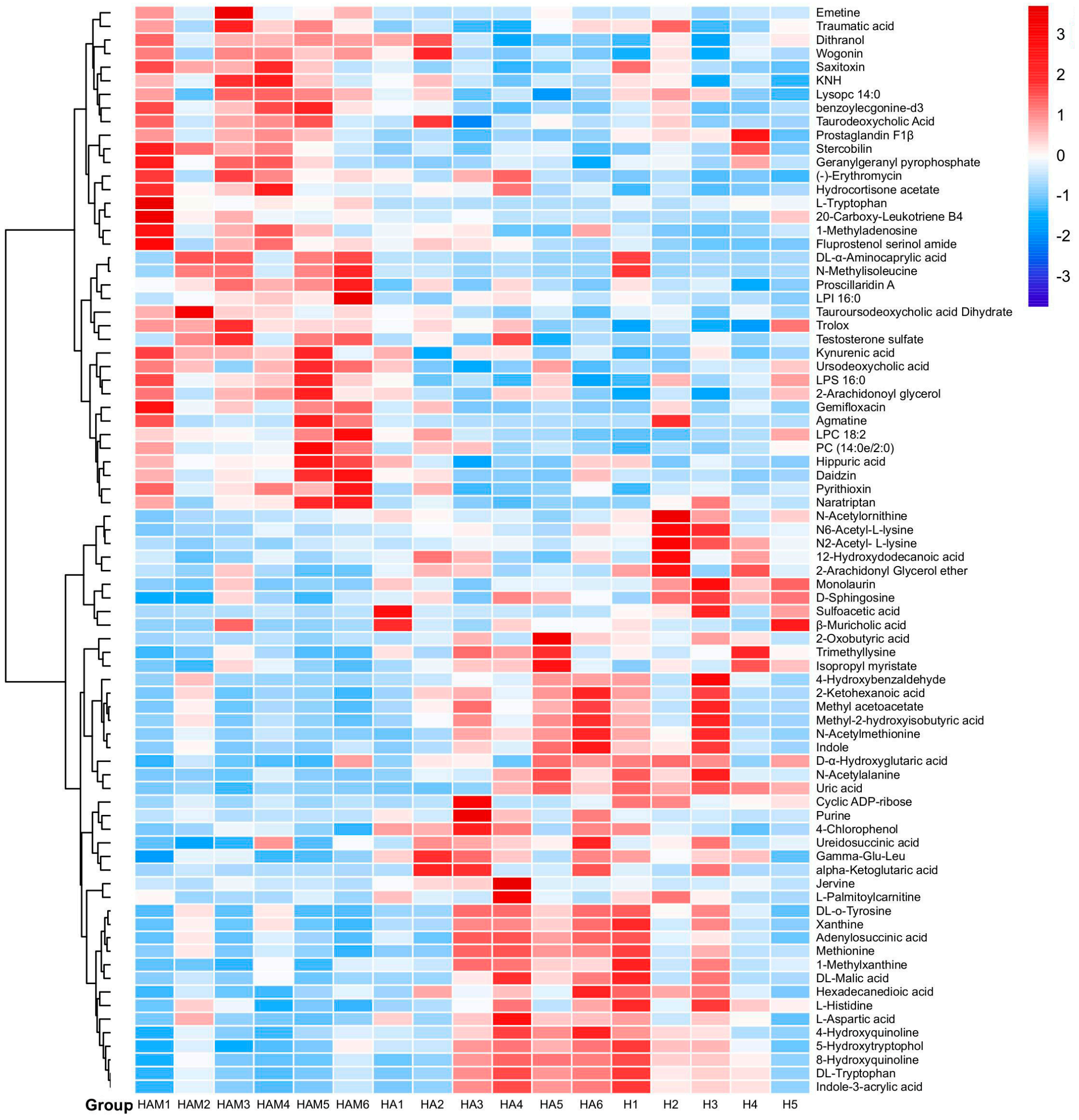

Additional file. Figure S4. Clustering heat map of differential metabolites in liver metabolites.

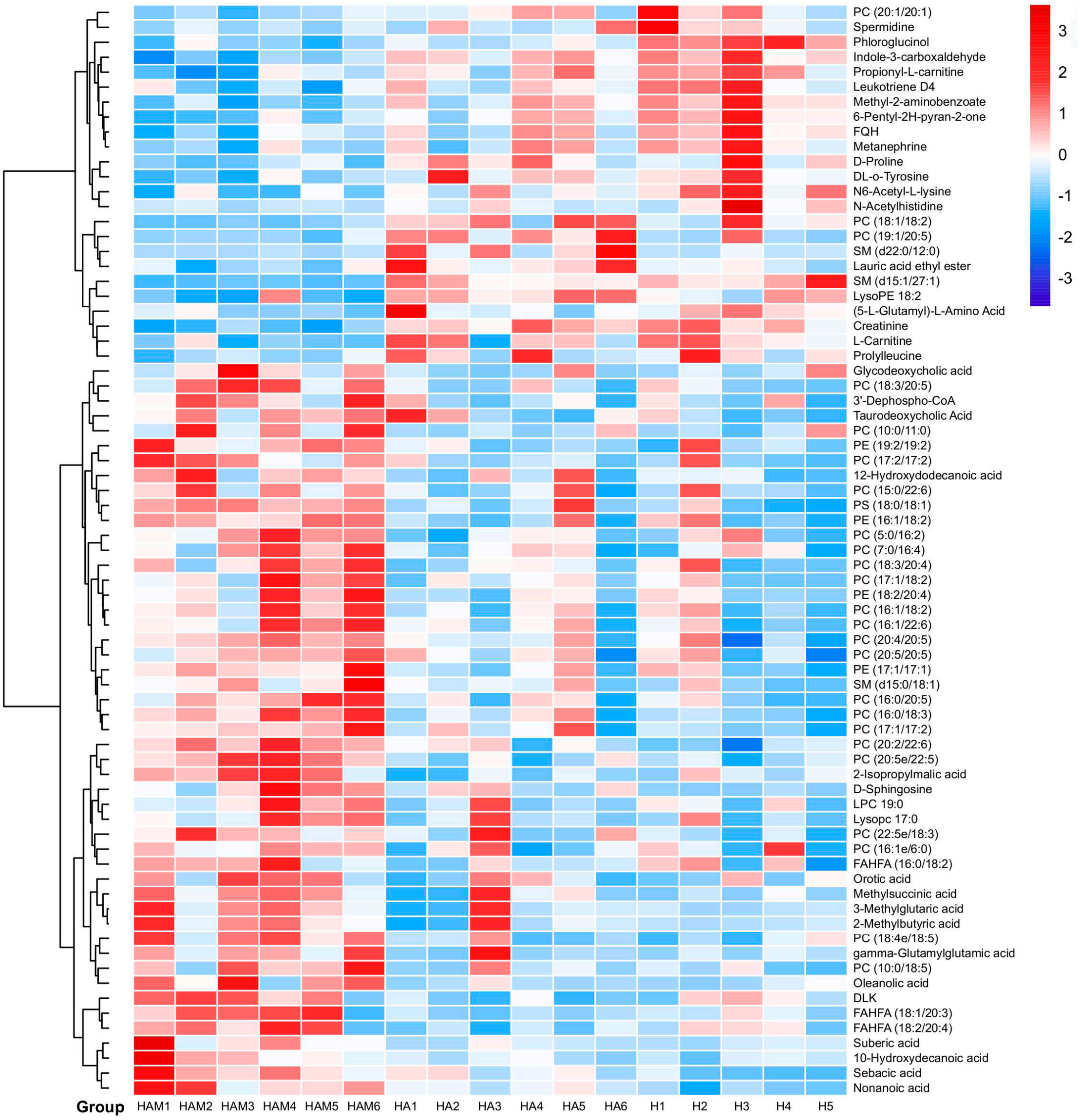

Additional file. Figure S5. (a) KEGG enrichment bubble plot of fecal metabolites in HAM vs. HA. (b) KEGG enrichment bubble plot of fecal metabolites HAM vs. H.

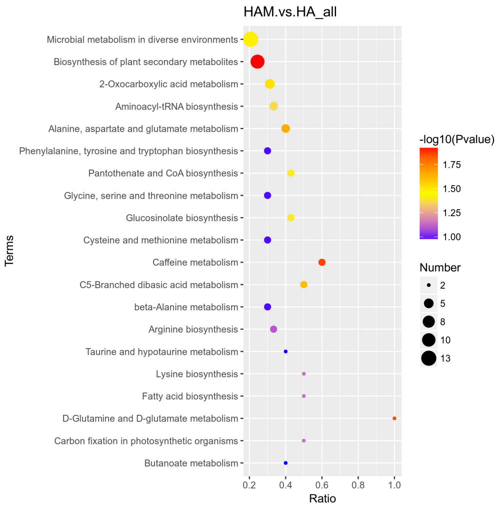

(a)

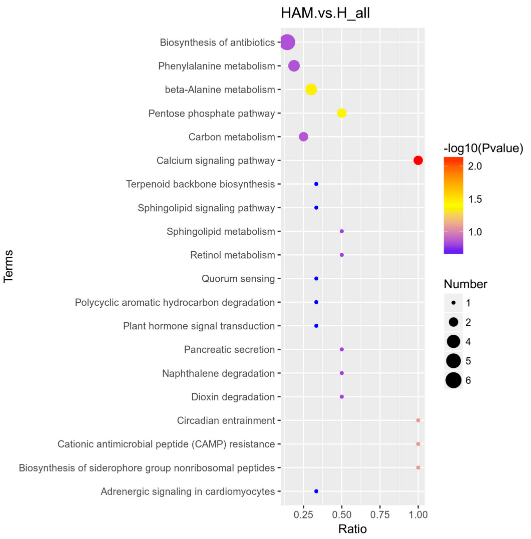

(b)

Additional file. Figure S6. (a) KEGG enrichment bubble plot of liver metabolites in HAM vs. HA. (b) KEGG enrichment bubble plot of liver metabolites in HAM vs. H.

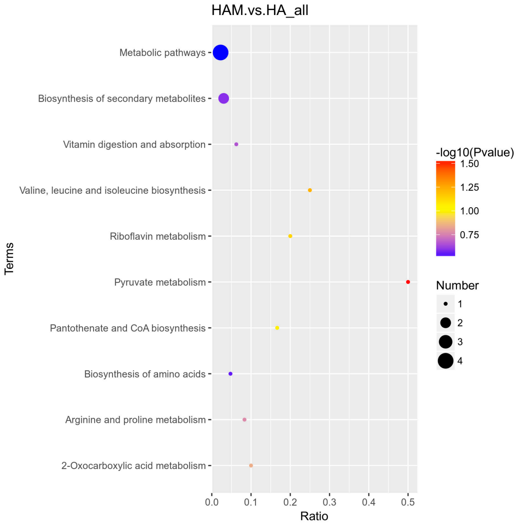

(a)

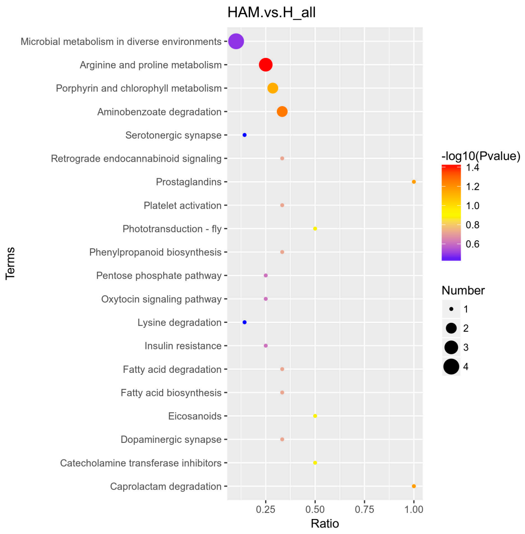

(b)

Additional file. Table S1.Relative abundance of gut microbiota at the phylum level.

Items	HAM	HA	H	*P*-Value
Bacteroidota	44.18±6.94	47.34±6.79	50.26±6.52	0.306
Bacillota	46.70±5.61	42.89±5.71	41.04±4.75	0.184
Unclassified bacteria	2.35±0.66	2.70±0.67	2.49±0.50	0.550
Pseudomonadota	0.54±0.19	0.80±0.57	0.68±0.62	0.556
Cyanobacteriota	0.18±0.09	0.20±0.10	0.44±0.51	0.198
Desulfobacterota	0.52±0.48	0.20±0.09	0.24 ±0.06	0.109
Elusimicrobia	0.20±0.12	0.09±0.04	0.16 ±0.08	0.062
Verrucomicrobiota	0.24±0.30	0.04±0.03	0.09 ±0.07	0.108
Methanobacteriota	0.00±0.00	0.01±0.02	0.00 ±0.00	0.564
Spirochaetota	0.00±0.00	0.01±0.01	0.00 ±0.00	0.426

Note:Data in the same column with different superscript letters indicate a statistically significant difference ($P < 0.05$). Data with the same or no letters indicate no statistically significant difference ($P > 0.05$).

Additional file. Table S2.Relative abundance of gut microbiota at the genus level.

Items	HAM	HA	H	*P*-Value
Bacteroides	4.60±1.63	4.62±1.22	4.11±1.58	0.803
Christensenellaceae_R-7_group	2.33±1.22[b]	4.60±2.58[a]	2.49±.81[ab]	0.046
Lactobacillus	3.36±1.85	2.28±2.01	2.97±1.02	0.486
Ruminococcus	2.91±0.85	2.64±0.60	2.14±0.45	0.172
Lachnospiraceae_NK4A136_group	2.34±0.66[a]	1.70±0.69[b]	1.45±0.26[b]	0.038
UCG-005	1.21±0.51	1.89±0.71	1.80±1.12	0.194
Ligilactobacillus	1.94±1.15	1.40±0.78	1.30±0.51	0.373
NK4A214_group	1.07±0.28[b]	1.67±0.40[a]	1.39±0.39[ab]	0.012
Romboutsia	1.27±0.48	1.31±0.34	1.61±0.47	0.358
Prevotella	0.20±0.22[b]	0.69±0.36[b]	4.09±4.78[a]	0.018
Colidextribacter	1.36±0.51	1.35±0.37	1.12±0.41	0.584
[Eubacterium]_siraeum_group	1.16±0.71	1.62±1.23	0.98±0.33	0.410
Intestinimonas	0.79±0.30	0.95±0.39	0.89±0.32	0.621
Oscillibacter	0.72±0.25	0.81±0.32	0.68±0.22	0.671
Prevotellaceae_UCG-001	0.80±0.31	0.71±0.38	0.63±0.33	0.680
Lachnospiraceae_UCG-001	0.79±0.46	0.58±0.33	0.61±0.36	0.504
Prevotella_9	0.12±0.14[b]	0.27±0.16[b]	1.71±2.34[a]	0.049
[Eubacterium]_xylanophilum_group	0.60±0.20	0.57±0.26	0.41±0.11	0.272
Alloprevotella	0.36±0.24	0.27±0.18	1.01±1.68	0.270
Parabacteroides	0.44±0.21	0.47±0.11	0.46±0.27	0.963
Limosilactobacillus	0.33±0.12[b]	0.38±0.11[b]	0.73±0.46[a]	0.024
Prevotellaceae_NK3B31_group	0.22±0.17	0.65±0.68	0.31±0.24	0.168
Alistipes	0.38±0.12	0.44±0.12	0.34±0.24	0.548
Parasutterella	0.36±0.12	0.43±0.11	0.37±0.16	0.594
Roseburia	0.33±0.21	0.46±0.26	0.35±0.05	0.419
[Eubacterium]_ruminantium_group	0.84±0.86[a]	0.15±0.18[b]	0.02±0.01[b]	0.024
Blautia	0.45±0.29	0.35±0.17	0.33±0.11	0.559
Monoglobus	0.36±0.18	0.45±0.15	0.29±0.08	0.229
Clostridium_sensu_stricto_1	0.34±0.23	0.31±0.08	0.51±0.31	0.235
Desulfovibrio	0.09±0.08[b]	0.50±0.49[a]	0.21±0.04[ab]	0.046

Note:Data in the same column with different superscript letters indicate a statistically significant difference ($P < 0.05$). Data with the same or no letters indicate no statistically significant difference ($P > 0.05$).
